# Respiratory Syncytial Virus Bronchiolitis in Infancy: The Acute Hospitalization Cost

**DOI:** 10.3389/fped.2020.594898

**Published:** 2021-01-18

**Authors:** Elena Bozzola, Claudia Ciarlitto, Stefano Guolo, Carla Brusco, Gennaro Cerone, Livia Antilici, Livia Schettini, Anna Lucia Piscitelli, Anna Chiara Vittucci, Renato Cutrera, Massimiliano Raponi, Alberto Villani

**Affiliations:** ^1^Pediatric and Infectious Diseases Unit, Bambino Gesù Children Hospital, Rome, Italy; ^2^Sanitary Direction, Bambino Gesù Children Hospital, Rome, Italy; ^3^Pneumology Unit, Bambino Gesù Children Hospital, Rome, Italy

**Keywords:** bronchiolitis, respiratory syncitial virus, cost, prevention, hospitaalization

## Abstract

**Introduction:** Respiratory syncytial virus (RSV) bronchiolitis is among the leading causes of hospitalization in infants. Prophylaxis with palivizumab may reduce RSV infection, but its prescription is restricted to high-risk groups. The aim of the study is to retrospectively determine acute hospitalization costs of bronchiolitis.

**Materials and methods:** Infants aged 1 month−1 year, admitted to Bambino Gesù Children Hospital, Rome, Italy, with a diagnosis of bronchiolitis from January 1 till December 31, 2017, were included in the study.

**Results:** A total of 531 patients were enrolled in the study, and the mean age was 78.75 days. The main etiologic agent causing bronchiolitis was RSV, accounting for 58.38% of infections. The total cost of bronchiolitis hospitalization was 2,958,786 euros. The mean cost per patient was significantly higher in the case of RSV (5,753.43 ± 2,041.62 euros) compared to other etiology (5,395.15 ± 2,040.87 euros) (*p* = 0.04).

**Discussion:** The study confirms the high hospitalization cost associated with bronchiolitis. In detail, in the case of RSV etiology, the cost was higher compared to other etiology, which is likely due to the longer hospitalization and the more frequent admission to the intensive cure department.

**Conclusion:** This study highlights that bronchiolitis is an important cost item even in a tertiary hospital and that cost-effective interventions targeting RSV are increasingly urgent.

## Introduction

Respiratory syncytial virus (RSV) is the most common viral cause of bronchiolitis (70%), with 3.4 million admissions and about 199,000 deaths per year in predominantly resourced-limited countries (1, 2).

In resource-rich areas, bronchiolitis is the main cause of hospitalization in the first 12 months of life, with an estimated cost in the USA of 500 million−1.7 billion dollars annually (3–6). In Australia and New Zealand, a recent review reported a population-based increase in admissions to the intensive care unit for bronchiolitis, with associated increases in hospital costs (7).

Previous studies suggest an increase of RSV-related costs, with increased odds of subsequent wheezing, respiratory-related hospitalizations, and higher healthcare resource utilization (8, 9).

The acute hospitalization cost (AHC) for bronchiolitis in Europe is about 2,000 euros per patient, with a considerable increase when pediatric intensive care unit (PICU) admission is needed (about 8,000 euros) (10).

Currently, no specific therapy is approved by any national guidelines, which recommend only supportive therapy that includes oxygen therapy for hypoxemia, respiratory support, and the maintenance of hydration (11, 12). Prophylaxis with palivizumab (a monoclonal antibody anti-F protein) reduces RSV infection and severity in the preterm population. However, the prescription of palivizumab is restricted worldwide and in Italy is limited to the group of <29 weeks of gestational age (wGA), age <12 months at the beginning of the RSV season, or group of <35 wGA with several risk factor (13, 14).

An Italian study highlights that most of RSV hospitalizations involved the pediatric population in the age class 0–4 years, with a peak in the first 12 months of life (15).

Although bronchiolitis is among the leading causes of hospitalization for infants, there are limited data concerning the epidemiology of bronchiolitis hospitalizations and the associated costs. The aim of the study is to retrospectively determine bronchiolitis AHC.

## Materials and Methods

For the purpose of the study, we included children aged 1 month−1 year, admitted to IRCCS Bambino Gesù Children Hospital, Rome, Italy, with a diagnosis of bronchiolitis.

The period study ranged from January 1 till December 31, 2017.

According to the NICE guidelines, bronchiolitis is defined as an acute respiratory illness in a child under 2 years of age, most commonly in the 1st year of life, characterized by coryza, persistent cough, and respiratory distress in the presence of wheezing or crackles during chest auscultation (16). The 2014 American Academy of Pediatrics clinical practice guideline on the diagnosis, management, and prevention of bronchiolitis describes bronchiolitis as “a constellation of signs and symptoms occurring in children younger than 2 years, including a viral upper respiratory tract prodrome followed by increased respiratory effort and wheezing” (11). For clinical research, bronchiolitis is typically defined as the first episode of wheezing in a child younger than 12 months who has physical findings of a viral lower respiratory infection and no other explanation for wheezing (17).

Accordingly, we excluded patients older than 1 year who may have other causes of recurrent wheezing that may require inhaled bronchodilators and chest radiography.

Bronchiolitis encounters identified by principal ICD diagnosis codes for bronchiolitis (ICD Diagnosis code 466.1, 466.11, 466.19, J21.0, J21.1, J21.8, J21.9).

Exclusion criteria comprised a detailed list of ICD-9 codes corresponding to multiple conditions and comorbidities that may complicate the course of treatment of these conditions. Patients with an ICD-10 code indicating asthma, sepsis, and pneumoniae are excluded from the population of patients with bronchiolitis. An additional exclusion criterion was a length of stay (LOS) of more than 10 days. According to All Patient Refined Diagnosis Related Group (3M Corp, Wallingford, CT), the four severity of illness subclasses and the four risk of mortality subclasses are numbered sequentially from 1 to 4 indicating, respectively, minor, moderate, major, or extreme severity of illness or risk of mortality. For any patient, we considered the highest severity level during hospitalization. As level 4 severity included the most critical cases with a high risk of death, we decided to exclude patients classified as level 4 from the study.

This way, the cases selected for the study have a short LOS, no other complications requiring intensive care, and no other chronic or comorbid conditions, such as neuromuscular or congenital heart diseases. Additionally, to minimize misclassification, for each category of respiratory conditions, we excluded cases with any of the other conditions if they were listed as a comorbidity because they may require different management. The resulting cohorts represent uniform populations of uncomplicated conditions.

Therefore, exclusion criteria were: age>12 months; previous episode of bronchiolitis; diagnostic code for asthma, sepsis, and pneumonia; LOS> 10 days, chronic or comorbid conditions as reported in diagnostic codes such as neuromuscular or congenital heart diseases, and patients classified as level 4 of All Patient Refined Diagnosis Related Group.

The medical records of patients with a principal discharge diagnosis ICD-9-CM code were analyzed in order to verify the clinical-confirmed diagnosis. Bronchiolitis was diagnosed clinically. A nasopharyngeal swab was prescribed to all infants with suspected bronchiolitis. Real-time PCR was performed on CFX96 (Bio Rad Laboratories, Italy) with a respiratory panel assay kit. The panel is made up of three mixes that allow the identification of 16 different viruses [Influenza A and B virus, Respiratory syncytial virus A and B, Adenovirus, Enterovirus, Parainfluenza virus 1, 2, 3, and 4, Metapneumovirus, Bocavirus, Rhinovirus, three Coronaviruses (NL63, 229E, and OC43), and three Influenza A subtypes]. An internal control was included in each sample to check both extraction efficiency and PCR inhibition. In every run, a negative control was used to monitor carry-over contamination. The results were analyzed automatically using Seegene software (Seegene Viewer V2.0).

Chest radiographs and other laboratory studies were not routinely performed; they were prescribed by the physician in case of worsening clinical conditions, suspected secondary or comorbid bacterial infection, or suspected complications.

Cost data were collected from the healthcare providers' perspective. As for the others, direct medical costs were extracted for each patient. Laboratory examinations include blood exams and nasopharyngeal swabs. As for imaging, some patients underwent a chest X-ray. Finally, treatment included supplemental oxygen, fluids for re-hydration, and other supportive treatment.

The appropriate procedure codes were applied to evaluate the single cost of any exam and therapy. The cost data were calculated using an Excel database reporting the cost for each patient correlated to laboratory and imaging exams, specialist evaluations (for example, pneumological or infectious consultant), therapy, and hospital accommodation. As regards hospitalization costs, they were calculated using the methodology developed in the national project “Mattone DRG,” which is applied to neonates and pediatric patients (18). In the Italian healthcare system, hospitalizations are remunerated according to a system that provides for the assignment of a standard remuneration (tariff), derived from a series of adjustments from the cost of the average hospitalization that refers to each DRG (Diagnosys Related Group), to each hospitalization. Each Italian region has its own tariff system. The coherence between the various regional remuneration systems is guaranteed by the presence of a reference national remuneration system from which the regions cannot deviate if for reasons agreed with the ministry. In this study, the Lazio Region Tariff Nomenclator DRG of outpatient services was used.

**Figure 1:**
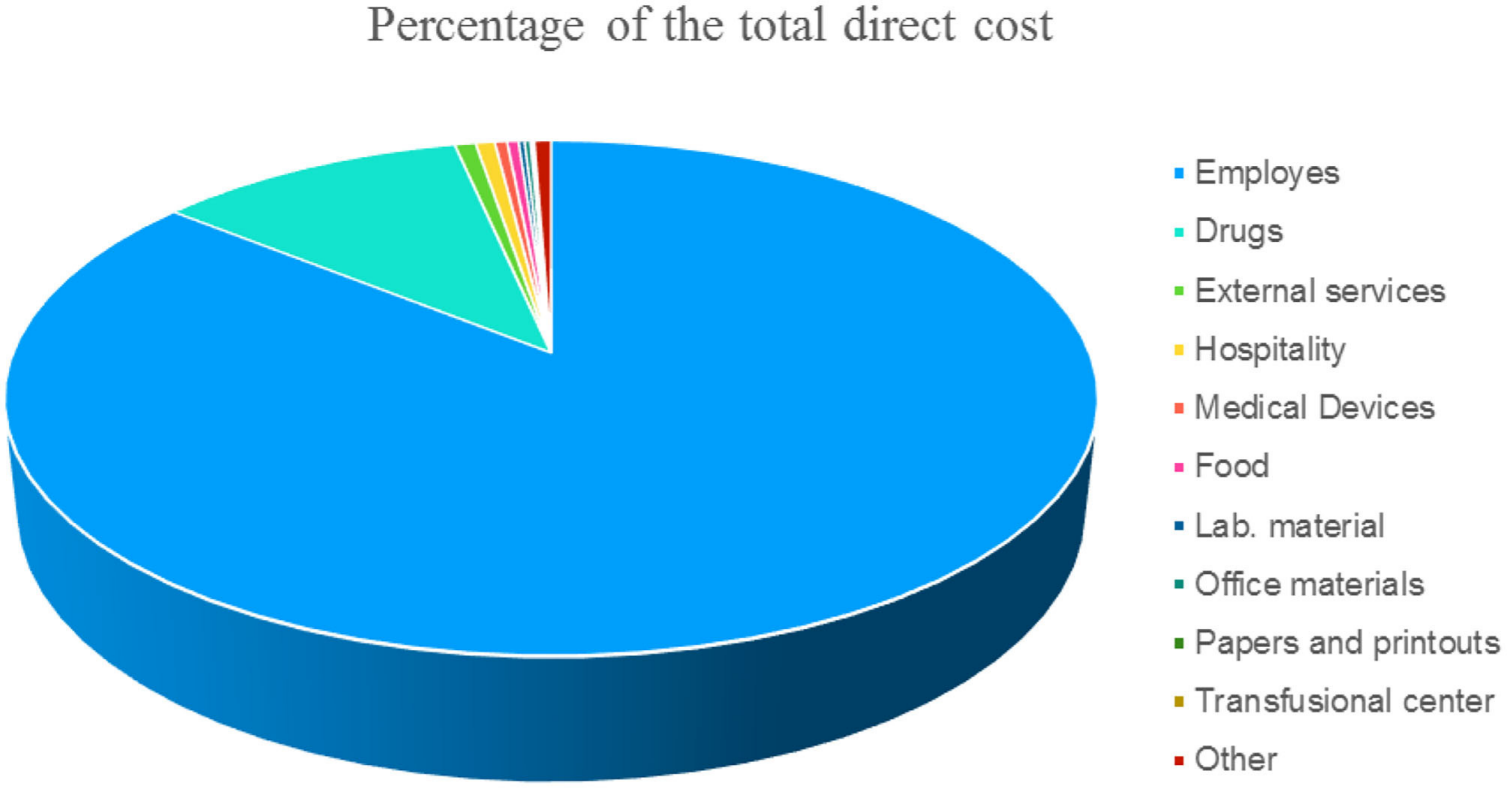
Items expressed in % of the direct cost (not attributable to the patient) of a single day of hospitalization.

This methodology provides the costing of hospitalization days on direct costs recorded at the cost center level. As the financial and administrative costs are very different between national, regional, and sometimes even local realities in order to allow for a comparison, we have indicated the items of direct costs that are independently from the patient in Figure 1 expressed as a percentage.

The total costs obtained from the direct cost (as divided in Figure 1) were then parameterized to the revenues from ordinary hospitalization to identify the portion of the costs attributable to the ordinary regime.

The total costs attributed to the ordinary regime were divided by the days of hospitalization, to determine the average cost per day of hospitalization. Then, the average cost had been calculated according on the length of hospitalization.

As regards the direct costs attributable to the individual patient, the average cost (in euros) per patient is shown in Table 1. As regards laboratory, imaging, consultations, drugs, outpatient tariffs, and the direct cost of drugs were used.

**Table 1:** Mean direct cost in euros attributable to the single patient based on the national remuneration system (Mattone DRG project).

**Item**	**Mean Cost**
Pediatric ward hospitalization	1,861.27
Picu hospitalization	1,510.98
Specialistic evaluation	25.43
Emergency department	287.74
Radiology	33.64
Operating room	–
Laboratory examination	3,392.22
Total mean cost	4,906.94

Costs were expressed in Euros.

Formal consent is not required for this kind of retrospective study: any personal data was protected accordingly to the Helsinki Declaration and Italian law. (Legislative Decree of 30 June 2003, n. 196. Code on the protection of personal data).

### Statistics

Data processing was performed with the Microsoft Excel 2018 and SPSS 20 software.

Comparison of categorical variables was done by the χ^2^ test. To test the statistical difference between means, a Student's *t*-test was used as appropriate. The level of statistical significance was set at 0.05.

## Results

Out of 601 patients hospitalized for bronchiolitis during 2017 at IRCCS Bambino Gesù Children Hospital, 70 were excluded as they did not fit the inclusion criteria. The sample size was 531 patients, 49.34% males, with a mean age of 78.75 ± 60.62 days.

**Figure 2:**
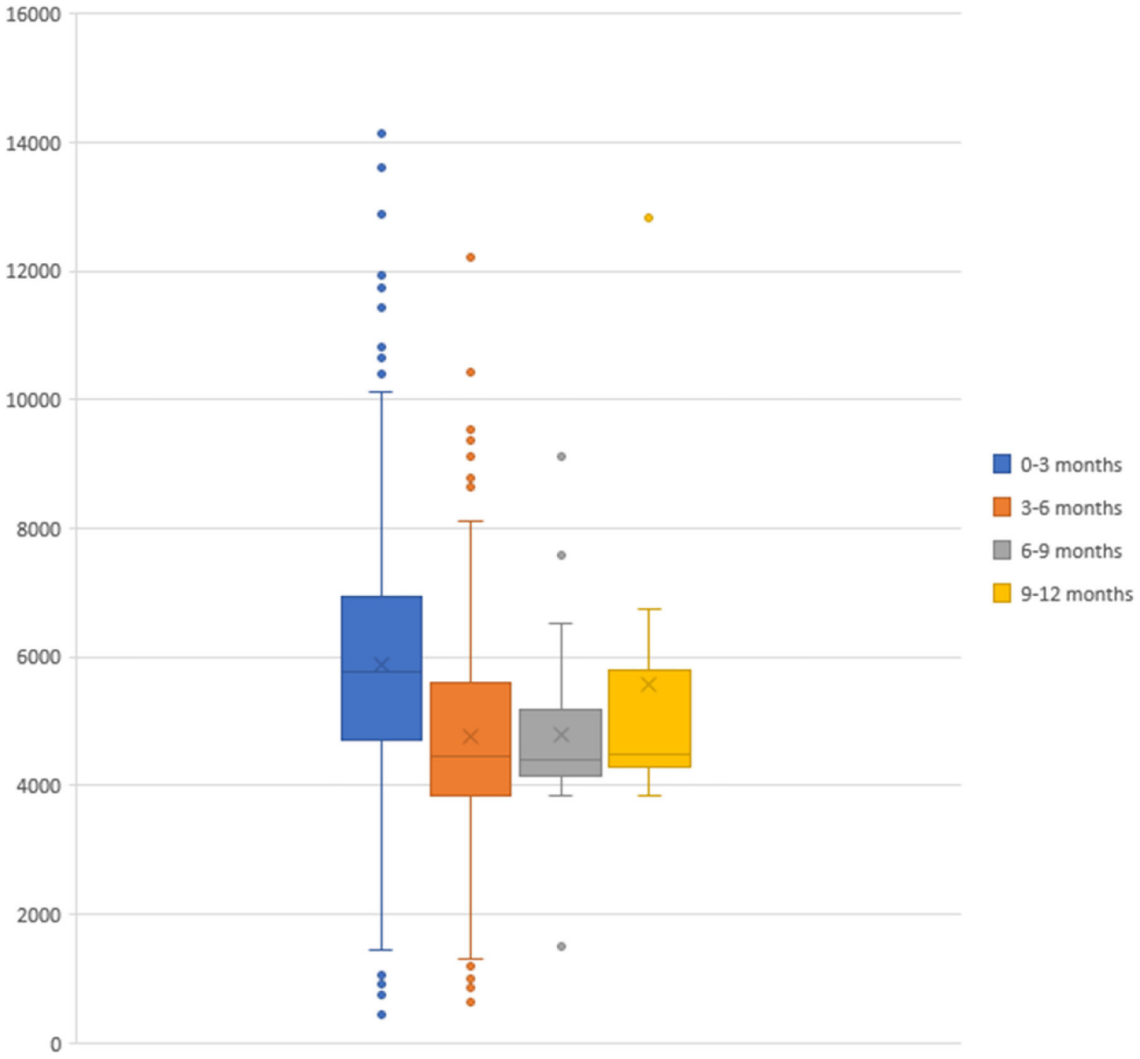
Acute hospitalization cost of bronchiolitis divided by age groups (expressed in euros).

The total cost of bronchiolitis hospitalization was 2,958,786 euros, which corresponded with a mean of 5,572.10 ± 2,037.79 euros per patient. Children < 3 months accounted for most of this annual cost, as is shown in Figure 2. Table 2 summarizes the characteristics of hospitalization in patients < or > 3 months of age.

**Table 2:** Demographic characteristics and AHC of patients < and > 3 months of age.

	**< 3 months**	**> 3 months**	***p*-value**
	***n* = 376**	***n* = 155**	
Males (%)	51.06	45.16	ns
LOS (days)	4.89 ±2.14	4.18 ± 2.29	0.001
Total cost (€)	2,210,570.56	748,215.37	
Mean cost (€)	5,879.18 ± 1,947.28	4,827.19 ± 2,066.18	<0.0001
Imaging (€)	18.36 ± 28.21	16.63 ± 27.18	ns
Laboratory (€)	3,574.60 ± 1,189.60	3,140.20 ± 1,138.60	<0.0001
PICU admission (%)	3.71	2.58	ns
RSV infection (%)	57.71	60	ns

*Data are shown as the number of cases (%) or mean (± SD)*.

The mean cost of bronchiolitis hospitalization was higher (5,572.10) than those calculated using the Lazio Region DRG's Tariffs (2,337.86).

The mean cost of a single patient accessing the ED (Emergency Department) was 286.35 euros. Of the 531 patients, 84.9% were treated with drug therapy in the ED, with a direct cost of 309.87 euros per patient.

**Figure 3:**
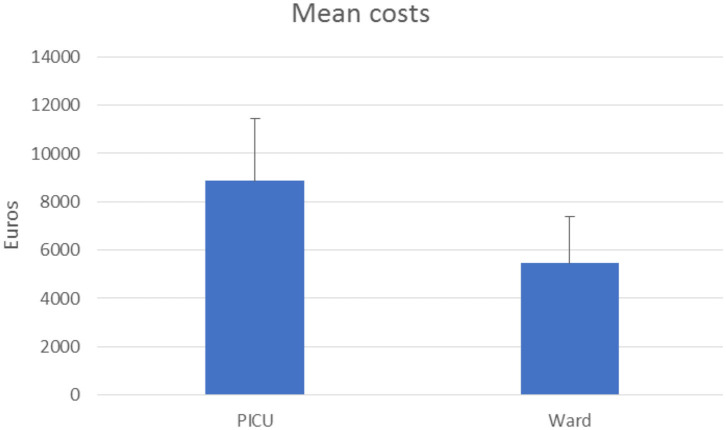
Acute hospitalization cost for bronchiolitis in a pediatric ward and in the PICU (expressed in euros).

A total of 3.39% of patients required intensive care with a mean cost of 8,859.99 ± 2,056.53 euros, which turns out to be about +161.5% of patients in a ward (5,456.74 ± 2,037.79 euros) as shown in Figure 3.

**Figure 4:**
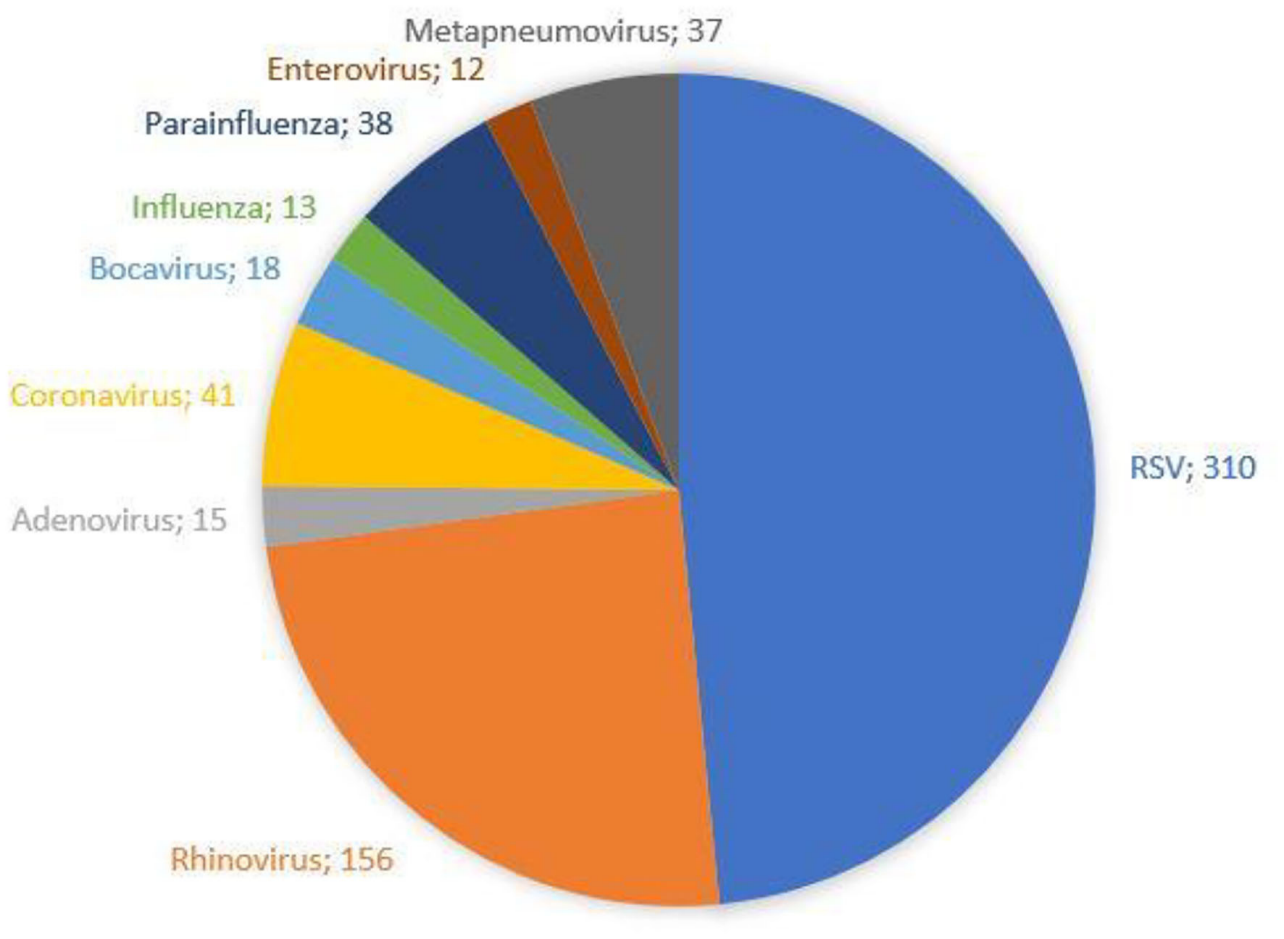
Bronchiolitis etiology: frequency of single viruses isolated by the Multiplex-PCR panel.

The demographic characteristics and AHC of patients admitted to PICU and pediatric wards are reported in Table 3. The main etiologic agent causing bronchiolitis was RSV, accounting for 58.38% of infections. Out of them, 41.24% of infants were affected by just RSV, while 17.14% were affected by RSV plus one more virus. Figure 4 summarizes bronchiolitis etiology in term of different viruses isolated by the Multiplex-PCR. A total of 23.72% of all patients were co-infected with two or more viruses, but the number of detected viruses did not correlate with any markers of severity (LOS, cost of hospitalization and interventions). In children affected by RSV, the LOS was longer than in those infected with other viruses (*p* < 0.001). The total cost for RSV related bronchiolitis was 1,783,562.76 euros, with a mean cost for each patient significantly higher in children infected with RSV compared to those who were not (p: 0.04). The mean cost per patient was significantly higher in the case of RSV (5,753.43 ± 2,041.62 euros) compared to other etiology (5,395.15 (± 2,040.87 euros) (*p* = 0.04). In fact, the RSV bronchiolitis course is generally more severe with a longer average duration of hospital stay and high oxygen requirement as well as increased requirement PICU. Table 4 summarizes the characteristics of hospitalization in patients with RSV infection. The data are related to all RSV infected children (*n* = 310). Out of them, 219 were only RSV while 91 wsere co-infected with other viruses. Among patients with RSV, we did not found significant differences between patients with RSV alone and patients with RSV plus another virus or more.

**Table 3:** Demographic characteristics and AHC in of patients admitted to PICU and pediatric ward.

	**PICU**	**Pediatric ward**	***p*-value**
	***n* = 18**	***n* = 513**	
Age (days)	51.17 (± 68.65)	79.72 (± 60.17)	0.049
Males (%)	66.67	48.73	Ns
LOS (days)	6.28 (± 1.60)	4.59 (± 2.20)	0.001
Total cost (€)	159,479.89 (± 2,056.53)	2,799,306.04 (± 2,037.79)	
Mean cost (€)	8,859.99 (± 2,577.61)	5,456.73 (± 1,919.52)	<0.0001

*Data are shown as the number of cases (%) or mean (± SD)*.

**Table 4:** Hospitalization data of children with Respiratory Syncytial Virus infection vs. other respiratory viruses infection.

	**RSV *n* = 310**	**Other *n* = 217**	***p*-value**
Age (days)	77.98 (± 58.02)	78.80 (± 64.16)	ns
Males (%)	45.8	54.83	ns
LOS (days)	4.98 (± 2.18)	4.22 (± 2.16)	<0.001
Total cost (€)	1,783,562.76	1,170,746.54	
Mean cost (€)	5,753.43 (± 2,041.62)	5,395.15 (± 2,040.87)	0.04
Imaging (€)	18.95 (± 28.62)	16.00 (± 26.41)	ns
Laboratory (€)	3,486.38 (± 1,126.19)	3,454.70 (± 1,204.50)	ns
PICU (%)	4.19	2.30	ns

*Among all patients (n. 531), four were not tested for respiratory virus infection. Data are shown as the number of cases (%) or mean (± SD)*.

## Discussion

This study confirms the high impact of bronchiolitis in terms of the cost of hospitalization in a tertiary care hospital. The total cost for uncomplicated bronchiolitis in children aged 1 month−1 year was 2,958,786 euros. The mean cost for AHC in pediatric wards was 5,456.73 euros. Meanwhile, for patients requiring intensive care, the cost was 8,859.99 euros (*p* < 0.0001) (Figure 3). The cost of hospitalization strongly correlates with the total LOS and the PICU stay. According to this result, a previous European study showed that the average hospitalization costs for <12 months old patients treated in PICU for bronchiolitis were more than four times higher than for those treated in a ward and over 20 times higher than for those treated in the ED (9). In our study, the total cost of radiological imaging (9,482.82 euros) and specialist evaluation for treatments (5,248.00 euros) did not effectively modify the average cost of hospitalization, which was mostly provided by the cost of hospital stay (908,931.69 euros) and of laboratory examinations (1,839,782.43 euros).

The mean direct cost of bronchiolitis admission in the ED was about 287 euros, which is consistent with the existing literature (19, 20).

The patients included in the study were mainly infants <3 months, and their admissions were those with a higher cost (Figure 2), as this age range has been identified as a risk factor for severe bronchiolitis (11). Therefore, the protection of this group of age could potentially prove to be essential to reduce the economic impact of bronchiolitis.

In our study, RSV was the main infective agent responsible for bronchiolitis, with a total cost of 1,783,562.76 euros and accounting for 72.22% of cases requiring PICU admission.

In children affected with RSV, the LOS was significantly longer (*p* < 0.001) and the mean AHC significantly higher (*p*: 0.04) compare to those who were not, as reported in Table 4. Moreover, in children with more than one virus detached during hospitalization, the number of detected viruses did not correlate with any markers of severity. Consequently, we can speculate that eliminating RSV may reduce AHC related to bronchiolitis.

The relevant role of RSV as the major etiologic agent of bronchiolitis was already described by an Italian group in 2014, and the burden of RSV-associated admission is confirmed by a prospective multicenter study performed in Spain, proving that costs were significantly higher in children positive to RSV (19, 20). Moreover, in a recent study, we reported an increase in PICU admission rates due to RSV-bronchiolitis (21).

In this study, 3.39% of patients required intensive care. Although in lower percentages than those reported in the literature, these cases have considerably higher costs and are mostly related to RSV. Therefore, the definition of predictive markers in severe bronchiolitis, and in particular in RSV, remains an urgent challenge. The absence of specific drugs against RSV makes the implementation of a vaccination strategy even more in demand, particularly to the age group of infants <3 months (22–24). Passive immunization with a humanized monoclonal antibody (IgG) directed against an epitope in the A antigenic site of the F protein of RSV (palivizumab) is used in the prevention of respiratory syncytial virus disease as it reduces the incidence of severe RSV bronchiolitis and the impact of long term-complication in premature infants (25). As reported by Coletta et al. the optimization of the use of palivizumab could minimize the cost of the drug and ensure the cost-benefit of prophylaxis (26). The Italian Drug Agency (AIFA) has restricted the eligibility for reimbursement to infants at high risk of hospitalization, ruling out palivizumab just in high-risk preterm infants. In our case report, only three children were eligible for palivizumab.

According to this study, a vaccination to protect infants < 12 months of age could be considered a potential strategy to reduce the health resources related to bronchiolitis.

The WHO Initiative for Vaccine Research identified the development of RSV vaccines as a priority (27). Studies are in progress on both vaccine and monoclonal antibodies, and a number of candidates are in development (28, 29). Infants <12 months of age are the target population with the greatest potential to benefit from the RSV vaccine, which should ideally be delivered in the first 6 months of life. As suggested by data from ongoing studies, also maternal immunization could be a good strategy to protect infants during their period of greatest vulnerability to RSV infection (30–32). This study has some limitations as it is retrospective and does not consider secondary and follow up costs. Finally, the overall costs of bronchiolitis in our organization has been underestimated due to the inclusion/exclusion criteria of patients.

## Conclusions

This study highlights that bronchiolitis is an important cost item even in a tertiary hospital and that cost-effective interventions targeting RSV are increasingly urgent.

Bronchiolitis requires a commitment of many economic resources and represents an expensive cost item even for patients suffering from mild to moderate forms. Moreover, the need for intensive care almost duplicates the necessary costs, and the definition of prognostic markers in severe bronchiolitis is therefore fundamental.

The main cost item is related to young infants, in particulate to those of age <3 months, and RSV continues to be the main causative agent of severe bronchiolitis.

Vaccination strategies are urgently needed, such as the extension of immunoprophylaxis to infants.

## Data Availability Statement

The raw data supporting the conclusions of this article will be made available by the authors, without undue reservation.

## Author Contributions

EB planned the study. CC, LA, and LS collected the data. SG, CB, and GC performed the economic evaluations. AP performed statistical analysis. AC and RC revised the literature. MR and AV were major contributors in writing the paper. All authors contributed to the article and approved the submitted version.

## Conflict of Interest

The authors declare that the research was conducted in the absence of any commercial or financial relationships that could be construed as a potential conflict of interest.
